# Motor Vehicle Accident Due to Homonymous Hemianopia: An Unusual Presentation of Vertebrobasilar Stroke

**DOI:** 10.7759/cureus.15092

**Published:** 2021-05-18

**Authors:** Anood Al Rawahi, Salim A Al Busaidi, Humaid Al Kalbani, Abdullah M Al Alawi

**Affiliations:** 1 Medicine, Oman Medical Specialty Board, Muscat, OMN; 2 Medicine, Sultan Qaboos University Hospital, Muscat, OMN; 3 Ophthalmology, Oman Medical Specialty Board, Muscat, OMN

**Keywords:** stroke, visual field, vertebrobasilar circulation, motor vehicle accident., homonymous hemianopia

## Abstract

Acute ischemic stroke may present with various symptoms, including weakness, altered speech, and sensory and visual impairment. We present a case of a 57-year-old man who was brought to the emergency department after he sustained three minor motor vehicle accidents on the same day. After clinical assessment and detailed workup, we concluded that our patient had an acute ischemic infarct involving the left posterior cerebral artery territories causing right homonymous hemianopia resulting in motor vehicle accidents.

## Introduction

Vertebrobasilar (VB) ischemia accounts for about 20% of all transit ischemic attacks and strokes [1]. VB ischemic symptoms include dizziness, unilateral limb weakness, dysarthria, headache, nausea, vomiting, nystagmus, and gait ataxia. The manifestations are because VB circulation is the main supply of the brain stem, cerebellum, and occipital cortex [2]. Homonymous hemianopia is the most common type of visual field defect seen following VB stroke. Other causes of homonymous hemianopia include head trauma, multiple sclerosis, brain tumor, and neurosurgical procedures [3]. We report a case of a patient who had motor vehicle accidents as the first manifestation of VB stroke.

## Case presentation

A 57-year-old man, known to have a poorly managed type 2 diabetes mellitus on oral hypoglycemic agents, was brought to the emergency department (ED) following three minor motor vehicle accidents happening the same day. He was driving back home from work when he had a sudden onset of blurred vision that resulted in a collision with the car ahead of him. He was driving at low speed, and there were minor damages to both cars but no injuries. Unfortunately, the patient continued his way back home and he hit another two cars sustaining minor motors damage, but there were no injuries. Upon arrival home, his family noted that he was confused, so they brought him to the ED. The patient described an episode of vomiting, visual disturbance, and right-sided weakness before the car accidents.

On clinical examination, he was confused and had nominal aphasia. His vitals were as follows: temperature 36.9°C, blood pressure 144/98 mmHg, heart rate 82 beats/minute, respiratory rate 19 per minute, oxygen saturation 99% on room air, and blood sugar level 24 mmol/L. The visual field examination revealed right homonymous hemianopsia. Also, the patient had a mild right-sided weakness (4/5) involving the upper and lower limb sensations, ataxic gait, and normal reflexes and sensation. Other systemic examinations were unremarkable.

**Figure 1:**
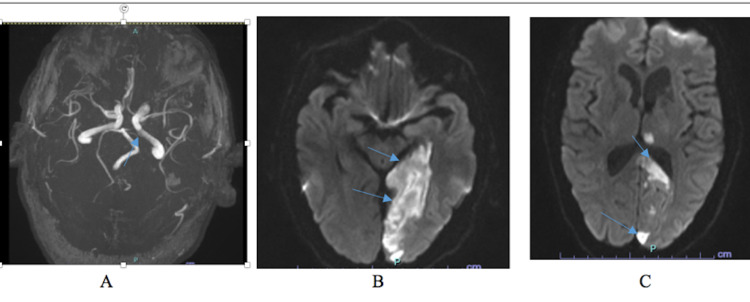
An MRA illustrating an acutely occluded left posterior cerebral artery (P1, occlusion from origination) (A) with acute ischemic infarct involving the majority of its territory including left posteromedial thalamus, left occipitotemporal part, splenium and a small part of above lying corona radiata in parieto-occipital (B and C).

Plain CT of the brain did not show any evidence of acute cerebral insult. Glycosylated hemoglobin was 14.3% (normal 4.5-5.7). Other investigations including full blood count, urea and electrolytes, vitamin B12 level, thyroid function test, lipid profile, bone profile, and electrocardiogram (ECG) were unremarkable. The brain magnetic resonance angiography (MRA) showed acutely occluded left posterior cerebral artery (PCA) with acute ischemic infarct involving the majority of its territories, including left posteromedial thalamus, left occipitotemporal part, splenium, and a small part of the ipsilateral corona radiate, and the parieto-occipital part as a watershed (Figure 1). Echocardiogram and 24-hour Holter electrocardiography were not suggestive of a cardio-embolic source of the stroke. An electroencephalogram (EEG) showed a mild intermittent slowing in the left temporal region, but no definitive epileptiform discharges were seen. 

The patient was admitted with the impression of acute ischemic stroke, and he was treated with clopidogrel, aspirin, and atorvastatin. He received insulin therapy for glycemic control. The patient's ophthalmological assessment showed a best-corrected visual acuity (BCVA) of 6/15 in the right eye and 6/7.5 in the left eye, normal intraocular pressure, normal extraocular movement. The confrontation test showed right temporal hemianopia. Slit-lamp biomicroscopy, funduscopy, Optical Coherence Tomography (OCT) of the macula, and Retinal Nerve Fiber Layer (RNFL) were unremarkable. Humphrey visual field 30-2 SITA Standard Test showed complete right homonymous hemianopia, which correlates with MRI brain lesion in ischemic infarct in the territory of left PCA affecting the left occipital region. 

The patient had allied health assessment and had sessions of inpatient physiotherapy. During the hospital stay, the patient showed improvement in terms of confusion, ataxia, right-sided weakness. However, he continued to have right-sided homonymous hemianopia. The patient was reviewed regularly in the outpatient department (OPD). He reported residual partial visual loss causing an inability to resume some of the important pre-morbid activities, including driving his car.

## Discussion

Stroke is a leading cause of disability worldwide [4]. VB strokes and transient ischemic attacks (TIAs) account for 20% of all strokes [1]. VB vascular circulation consists of the vertebral, basilar, and posterior cerebral arteries and their branches [2,5]. Due to the large cerebral territories supplied by the VB artery, VB ischemic can present with a wide range of symptoms [2]. Causes of VB ischemia include atherosclerosis, cardioembolism, vertebrobasilar dolichoectasia, and arterial dissection [6].

Our patient had a history of poorly controlled diabetes mellitus, and with his presentation of new weakness and visual disturbance, ischemic stroke was the likely diagnosis. On the same day of admission, the patient had a history of motor vehicle accidents, so we considered traumatic brain injury as a differential diagnosis. However, weakness and visual disturbance proceeded the accidents. Primary ophthalmological condition (e.g., retinal detachment) could not explain the right-sided weakness and ataxia. After a detailed clinical examination and assessment, we concluded that our patient suffered from VB ischemic stroke resulting in motor vehicle accidents. MRI of the brain showed occlusion of the left posterior cerebral artery (PCA) with acute ischemic infarct involving most of its territories, thus explaining the patient's symptoms, including ataxia, homonymous hemianopia (HH), and weakness.

Homonymous hemianopia is a binocular, unilateral visual loss that affects the contralateral visual field due to unilateral damage to the visual pathway posterior to the chiasm, which is commonly caused by stroke [3,7]. HH can affect the ability to perform important daily activities including reading, driving, and navigation [3]. Driving is the primary mode of transportation in many countries [8]. HH impairs steering, lane positioning, and object detection on the blindside, hence explaining motor vehicle accidents. In many countries, people with HH are prohibited from driving [8-10]. Special eyeglasses and scanning training may improve the detection performance of drivers with HH [4,8]. Natural recovery occurs within the first month after stroke in 50% of patients with stroke, with minimal recovery occurring after six months [4]. Besides the optimization of diabetic management, physiotherapy, and ophthalmology follow up, we recommended a comprehensive fitness-to-drive assessment to ensure the patient's ability to drive safely. 

## Conclusions

Vertebrobasilar ischemia could present with a wide range of symptoms due to the large cerebral territories supplied by vertebrobasilar circulation. Homonymous hemianopia is commonly caused by stroke and can lead to devastating impairment of quality of life, including driving and reading impairment. Natural recovery of homonymous hemianopia occurs in about 50% of patients within the first one month following a stroke. However, a deficit that persists beyond six months is unlikely to recover. Patients with homonymous hemianopia must have a comprehensive fitness-to-drive assessment to ensure patients' and others' safety.
